# Rapid identification and interpretation of gene–environment associations using the new R.SamBada landscape genomics pipeline

**DOI:** 10.1111/1755-0998.13044

**Published:** 2019-06-24

**Authors:** Solange Duruz, Natalia Sevane, Oliver Selmoni, Elia Vajana, Kevin Leempoel, Sylvie Stucki, Pablo Orozco‐terWengel, Estelle Rochat, Susana Dunner, The NEXTGEN Consortium, The CLIMGEN Consortium, Michael W. Bruford, Stéphane Joost

**Affiliations:** ^1^ Laboratory of Geographic Information Systems (LASIG), School of Architecture, Civil and Environmental Engineering (ENAC) Ecole Polytechnique Fédérale de Lausanne (EPFL) Lausanne Switzerland; ^2^ Departamento de Producción Animal, Facultad de Veterinaria Universidad Complutense de Madrid Madrid Spain; ^3^ Department of Biology Stanford University Stanford California; ^4^ School of Biosciences Cardiff University Cardiff, Wales UK

**Keywords:** gene‐environment association, landscape genomics, Lidia cattle breed, local adaptation, Moroccan sheep, r‐package

## Abstract

samβada is a genome–environment association software, designed to search for signatures of local adaptation. However, pre‐ and postprocessing of data can be labour‐intensive, preventing wider uptake of the method. We have now developed R.SamBada, an r‐package providing a pipeline for landscape genomic analysis based on samβada, spanning from the retrieval of environmental conditions at sampling locations to gene annotation using the Ensembl genome browser. As a result, R.SamBada standardizes the landscape genomics pipeline and eases the search for candidate genes of local adaptation, enhancing reproducibility of landscape genomic studies. The efficiency and power of the pipeline is illustrated using two examples: sheep populations from Morocco with no evident population structure and Lidia cattle from Spain displaying population substructuring. In both cases, R.SamBada enabled rapid identification and interpretation of candidate genes, which are further discussed in the light of local adaptation. The package is available in the r CRAN package repository and on GitHub (github.com/SolangeD/R.SamBada).

## 1 INTRODUCTION

Local adaptation implies the existence of advantageous alleles conferring a population living in its native habitat a higher fitness than any other allochthonous population living in the same habitat (Kawecki & Ebert, 2004). Landscape genomics methods (Joost et al., 2007), including genome–environment association (GEA), are among the approaches used to detect signatures of local adaptation and have become increasingly popular, mainly due to the decreasing cost of sequencing, but also because of the recent availability of fine‐scale environmental data sets (Balkenhol et al., 2019; Rellstab, Gugerli, Eckert, Hancock, & Holderegger, 2015). However, the massive amount of data that can be analysed due to these improvements have made the development of more efficient tools essential (Stucki et al., 2017).

To this end, samβada was developed to perform large amounts of logistic regressions between genetic markers and multiple environmental variables (Stucki et al., 2017). samβada computes uni‐ or multivariate models between a binary genetic variable (e.g., the presence/absence of a genotype) and one or more environmental variables. Significance is assessed against a null model (i.e., constant model in the case of univariate or a parent model in the multivariate case). Population structure can be accounted for by treating one or several population variables as environmental variables in multivariate analysis. samβada is written in C++ with a particular emphasis on high‐performance computing (HPC). Since its publication, samβada, as applied alone or in combination with other methods, proved useful to target putative genomic regions underlying local adaptation in a wide variety of species, including domestic animals such as swine and cattle (Cesconeto et al., 2017; Vajana et al., 2018), wild animals such as the freshwater sculpin and European pond turtle (Lucek, Keller, Nolte, & Seehausen, 2018; Pereira, Teixeira, & Velo‐Antón, 2018), and many different plant species including the European beech and the cow‐tail fir (Cuervo‐Alarcon et al., 2018; Shih, Chang, Chung, Chiang, & Hwang, 2018).

Despite its many advantages, samβada's command‐line format is sometimes laborious and the amount of pre‐ and postprocessing represents an obstacle to its widespread use. Indeed, a typical processing chain, such as the one proposed by Stucki et al. (2017), includes (a) the use of a GIS software to retrieve environmental information at sampling locations; (b) molecular data filtering by standard software such as plink (Chang et al., 2015); and (c) the inclusion, whenever present, of population structure usually computed with a dedicated software such as admixture (Alexander, Novembre, & Lange, 2009). Similarly, postprocessing of results involves (a) the computation of *p*‐ or *q*‐values (Storey, 2003) for the association tests involving each genotype; (b) the production of maps and plots (typically Manhattan plots) in which the location in the genome (i.e., the position in base pair) of a point representing the result of a model is difficult to establish since the plot is rarely interactive; (c) the formulation of queries to the Ensembl genome browser (Hubbard et al., 2002) to search for candidate genes adjoining the single‐nucleotide polymorphisms (SNPs) highlighted.

However, the r software (R Core Team, 2018) provides an open‐source computing environment adapted to different fields in Biology, in which many of the above‐mentioned pre‐ and postprocessing tasks can be found in various r‐packages. Further, r can be coupled with compiled languages (such as C++) so as to be more efficient when processing large data sets (see e.g., the case of the software LFMM 2; Caye, Jumentier, Lepeule, & François, 2019, p. 2).

In this context, we developed R.SamBada, an r‐package designed to facilitate and enhance the whole data process described above by integrating multiple existing packages and building new functions into one easy‐to‐use pipeline. We present the use of the package by illustrating its benefits with two case studies for which driven signatures of selection were investigated as part of the ClimGen project (https://climgen.bios.cf.ac.uk/). The first data set consists of 160 Moroccan sheep genotyped with whole genome sequencing (WGS) and characterized by no clear population structure, while the second one encompasses a Spanish Lidia Cattle population of 349 samples genotyped with 50 K SNP chip, with one population variable. Both data sets are already published (see Data availability section) but have not yet been analysed with samβada.

## 2 MATERIALS AND METHODS

We first present R.SamBada, with an overview of its functions, and then describe its application to two case studies from the ClimGen (https://climgen.bios.cf.ac.uk/) project, detailing how the genetic data were collected and prepared for subsequent analyses. Both studies investigate climate‐mediated selection at the genome level: the first analysis is carried out on a Moroccan sheep data set using whole genome sequences, and the second one involves a Spanish cattle breed (Lidia) genotyped with the Illumina BovineSNP50 array.

### 2.1 Implementation

**Figure 1:**
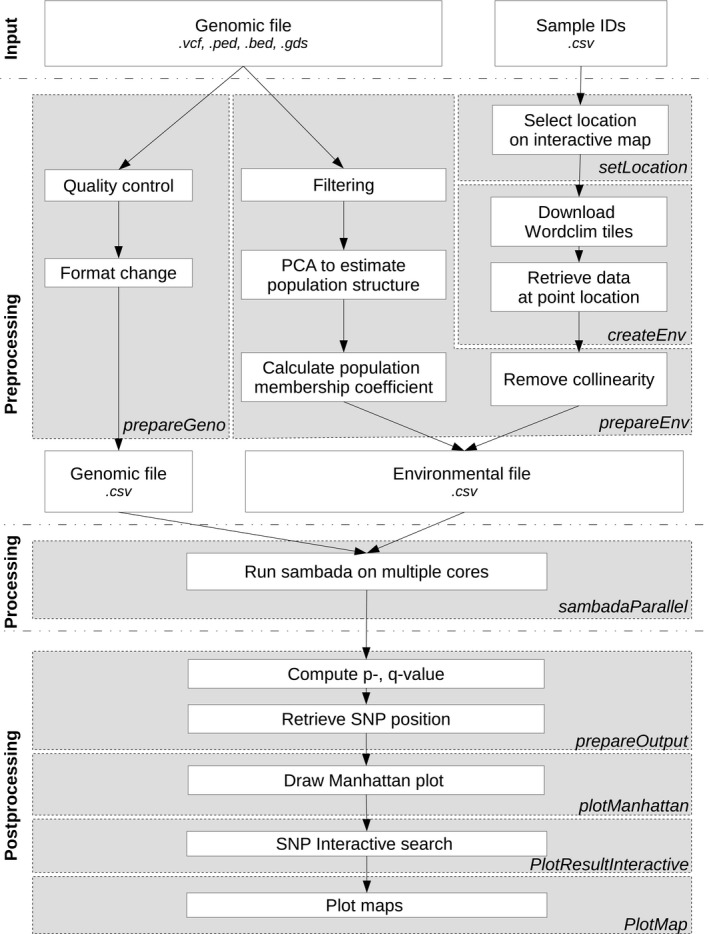
Overall functionalities and process in R.SamBada. Grey boxes with italic names indicate functions included in the package. The process starts with a genomic file and a file with sample locations or list of IDs. The preprocessing will format the genomic file and prepare the environmental file; samβada is then run parallelly on multiple cores; after computing of *p*‐, *q*‐values, Manhattan plots and maps can be drawn and Ensembl database can be queried

R.SamBada provides functions for (a) preparing the genetic (i.e., SNPs) and environmental information to be processed (preprocessing), (b) running samβada directly into the r environment (processing) and (c) performing post hoc analyses on the basis of samβada's output (postprocessing). The following sections detail these different steps (Figure 1).

#### 2.1.1 Preprocessing

Three functions have been implemented to perform the main operations required before running samβada. First, *prepareGeno* is used to prepare the genomic file, by treating a SNP input data set from various formats (.vcf, .gds, .ped or .bed) and generating a filtered file complying with samβada's input standards. *prepareGeno* relies on the snprelate package (Zheng et al., 2012) to perform standard quality control (QC) for minor allele frequency (MAF), linkage disequilibrium (LD) and missingness. In order to assist users in selecting adequate pruning levels, *prepareGeno* displays the frequency distributions of MAF, LD and missingness along with the proportion of SNPs discarded corresponding to the thresholds applied; in this way, QC can be tailored to avoid reducing the data set too much while controlling for missing information.

Second, if coordinates are not available, *setLocation* can be used to open a local web page that assist users in defining sample locations using mouse‐clicks on an interactive map. The projection system used is WGS84 (corresponding EPSG – European Petroleum Survey Group – code: 4326), a worldwide system with coordinates in degrees (longitude/latitude) (more information on projections in Leempoel et al., 2017).

Then, *createEnv* provides the user with a pipeline to produce an environmental data set out of the file containing sample locations. If raster files representing environmental variables are available, then habitat information is directly derived at the sampling locations. However, if these files are not present, *createEnv* is able to use the samples’ geographic coordinates to identify the correct tiles in the WorldClim (Hijmans, Cameron, Parra, Jones, & Jarvis, 2004) and SRTM (Shuttle Radar Topography Mission) (Farr et al., 2007) databases and to download adequate climatic and altitudinal information. The WorldClim database contains monthly minimum, maximum and average temperature and total precipitation together with a series of bioclimatic variables computed from these variables (e.g., precipitation of wettest quarter of the year, complete list available at http://www.worldclim.org/bioclim), while SRTM only provides altitude. Coordinates can be given in any projection system (as long as the EPSG code of the projection is given as an input parameter of the function). A comma‐separated value (.csv) file is then returned containing the sample IDs, their locations and the values of the corresponding environmental variables. The interactive mode shows maps of sample locations, so as to locate potentially misplaced points or erroneously‐set projection systems. This function can save substantial effort, since one single command substitutes a long processing chain that typically includes the download of voluminous data for the entire globe, the import of both sample locations and raster environmental data into GIS software and the retrieval of environmental values at point location.

Finally, the *prepareEnv* function produces a file containing the design matrix that samβada will process. At first, highly correlated environmental variables are removed according to a correlation coefficient threshold defined by the user in order to keep only independent eco‐climatic factors in the analysis. The interactive mode will show the graph of the number of variables discarded as a function of the chosen correlation threshold. Then, the genetic structure of populations is assessed by means of a principal component analysis (PCA) as implemented in snprelate. The user is provided with the possibility of further processing PCA output by a clustering algorithm, which calculates individual membership coefficients as a function of the distance from the clusters centroids (Lee, Abdool, & Huang, 2009). Changes in the clustering solution according to the chosen k‐number of clusters can be interactively visualized. After ordering individuals according to their identifiers (as in the genomic file and necessary for samβada's analysis), a final.csv file is generated, containing the samples’ IDs, the retained environmental variables and either the PCA score(s) or the membership coefficient(s) representing population structure.

#### 2.1.2 Processing

samβada includes a useful module called supervision that is designed to split the input file into several subfiles and merge the split result files, thus reducing drastically the computation time by allowing manual start of parallel sessions. This module has however rarely been employed to date, possibly due to its laborious and time‐demanding preparation procedure. This limitation is overcome in R.SamBada through the *sambadaParallel* function that implements supervision by default, and relies on the doparallel r‐package (Microsoft Corporation & Weston, 2017). Furthermore, unlike the previous version of samβada (0.5.1 used in Stucki et al., 2017), version 0.8.1 (included in R.SamBada) makes it possible to directly assess the effect of population structure by comparing the full model (containing all population variables and one or more environmental variables) with the null model (containing only population variables).

#### 2.1.3 Postprocessing

Four ad hoc functions have been developed for obtaining and visualizing samβada's outputs. In the postprocessing pipeline, the statistical significance of genotype–environment associations is derived since only G‐ and Wald‐scores are calculated by samβada, and no hypothesis testing is performed. Here, R.SamBada provides the function *prepareOutput*, which computes (i) *p*‐values by comparing the spread of G‐ or Wald‐scores from samβada to a chi‐squared distribution and (ii) *q*‐values based on Storey's method (Storey, 2003). The visualization of the position of outlier loci along the genome is possible using the *plotManhattan* function that generates Manhattan plots based on the *p*‐ or *q*‐values as computed by *prepareOutput*.

Next, *plotResultInteractive* can be used to display interactive Manhattan plots. In particular, users can specify which chromosome(s) they want to visualize for which environmental variable, the *p*‐ or *q*‐values, being then plotted for each genotype as a function of their genomic coordinates. Marker name, position, *p*‐value, functional relevance (e.g., intergenic‐, nonsynonymous variants) as well as proximal genes – whenever present – can be then retrieved for each marker by directly clicking on the set of points of interest being displayed. Gene annotation and functional investigation are performed by internal calls to the Ensembl genome browser (Hubbard et al., 2002) and the Variant Effect Predictor (VEP) (Yates et al., 2015), respectively, while the whole interactive graphical interface relies on the r‐package shiny (Chang, Cheng, Allaire, Xie, & McPerson, 2018). Additionally, a basic geographic map shows the geographic distribution of the marker, the environmental variable and the population structure (examples presented in Figure S1).

Finally, the *plotMap* mapping function makes it possible to represent the geographic distribution of (a) the putative signature(s) of selection, (b) the environmental pressure associated (as a raster background if available), (c) the neutral population structure (Figure 5 for an example) and (d) the degree of genetic similarity among sampling sites for the target markers (i.e., its spatial autocorrelation, see Stucki et al., 2017). *plotMap* relies on the functionalities embedded within the packcircles r‐package (Bedward, Eppstein, & Menzel, 2018) to shift nearby sampling points and prevent them from overlapping.

### 2.2 Case studies

#### 2.2.1 Moroccan sheep

##### Sampling and genetic data

Moroccan sheep (*Ovis aries*) populations constitute an excellent case study to investigate potential local adaptation through landscape genomics, since they have experienced (a) low anthropogenic selective pressure (Guessous, Boujenane, Bourfia, & Narjisse, 1989) and (b) contrasted climatic conditions throughout the whole country, as imputable to presence of the Atlantic Ocean, the Atlas Mountain and the Saharan desert in the south. WGS data from sheep populations in Morocco were produced and made available by the NextGen project (https://nextgen.epfl.ch) and are analysed for climatic selection signatures in the present study. A total of 164 individuals were sampled according to a grid composed of 162 cells of 0.5° of longitude/latitude each, so as to maximize the range of environmental conditions and geographic distribution (Figure 3). Detailed sequencing and genotyping information are described in Alberto et al. (2018).

##### Preprocessing

Quality control analysis was performed using the *prepareGeno* function with MAF <0.05 and SNP missingness <0.1, leading to a pruned data set composed by 20,226,452 SNPs (corresponding to 60,679,356 genotypes). SRTM and Worldclim variables (56 in total) were downloaded with *createEnv*, and *prepareEnv* was run to check for variable correlation in order to exclude variables showing an *r* ^2^ higher than 90%, resulting in a final data set consisting of 16 environmental variables (13 Bioclim variables, 2 raw WorldClim and altitude). No population variable was included in samβada's models (univariate mode) since no evidence of population structure emerged using the PCA method implemented in snprelate (with genomic filter of MAF <0.05, SNP missingness <0.1 and LD threshold <0.2).

##### Postprocessing

*q*‐values based on G‐scores were visualized with a Manhattan plot using a significance threshold of 0.05. *plotResultInteractive* was used to detect genes neighbouring the markers under selection as well as to identify variant functions (e.g., nonsynonymous SNPs).

### 2.3 Spanish Lidia cattle

#### 2.3.1 Sampling and genetic data

The Lidia cattle breed (*Bos taurus*) emerged during the XVIII century and evolved mainly in the *dehesas* ecosystems of the west/south‐west Iberian Peninsula, composed of pasturelands interspersed with Mediterranean oaks (*Quercus ilex*) (del Barrio, Ponce, Benavides, & Roig, 2014). Since its establishment, Lidia was prompt to isolation by preventing crossbreeding with allochthonous cattle (Eusebi, Cortés, Dunner, & Cañón, 2017) and became fragmented into reproductively isolated lineages (called *encastes*) with homogeneous morphology, behaviour and genetics (Boletin Oficial del Estado, 2001). Such a peculiar evolutionary and cultural context boosted Lidia's population size to become the largest Spanish breed and made it one of the most inclusive intergrading bovine population, granting high level of genetic richness among *encastes* coupled with low average genetic diversity values within lineages (Cañón et al., 2008). A total of 349 individuals were sampled among 61 different breeders evenly distributed across southern Spain's *dehesas* region (Figure 4). Between one and seventeen animals per breeder were selected based on pedigree information to minimize the risk of kinship among individuals. Animals were genotyped using the Illumina BovineSNP50 array v.2 (Eusebi et al., 2017).

#### 2.3.2 Preprocessing

Quality control analysis was performed using the *prepareGeno* function with a MAF <0.05 and SNP missingness <0.1. The resulting molecular data set consisted of 38,335 SNPs (i.e., 115,005 genotypes). SRTM and Worldclim variables (56 in total) were downloaded with the *createEnv* function, and *prepareEnv* was used to test for variable correlation resulting in only 15 variables (10 Bioclim and 5 raw WorldClim variables) kept which showed a *r* ^2^ lower than 90%. Due to the presence of population structure observed with snprelate's PCA method (see Results section), samβada was run in bivariate mode by adding a variable to account for population structure (score of the first PCA). This variable is not correlated with other kept environmental factors (highest correlation: precipitation in April, *r* ^2^ = 0.25).

#### 2.3.3 Postprocessing

*p*‐values based on *G*‐Scores were corrected for multiple testing with Bonferroni method and subsequently were displayed in a Manhattan plot (*q*‐values were not conservative enough in that case), with a significance threshold of 0.05, and *plotResultInteractive* was then used to detect associated genes.

## 3 RESULTS

### 3.1 Time efficiency

Besides the time saved during pre‐ and postprocessing, R.SamBada is more time‐efficient than using samβada's command line (version 0.5.1) for two reasons: first, R.SamBada automatically integrates supervision to distribute the processing of models over several cores, which makes the analysis run *x* times faster (where *x* represents the number of CPU), to which we must add a few minutes to split and merge the data set (e.g., 24 min to split and merge the sheep data set, compared to 160 hr saved by parallel computing on the same 11 cores). Second, if population variables are included in the analysis, the new version of samβada (0.8.1) will only focus on models including population variables. Here, the time saved will depend on the number of population variables (for the Lidia cattle analysis, with one population variable, it reduced the computing time from 53 to 9 min).

### 3.2 Moroccan sheep

#### 3.2.1 Population structure

The variance explained by the first three PCA components was 0.0085, 0.0083 and 0.0082, respectively, indicating no clear population structure. Therefore, no variable translating population structure was retained for subsequent analyses.

#### 3.2.2 Genotype‐environment associations

**Figure 2:**
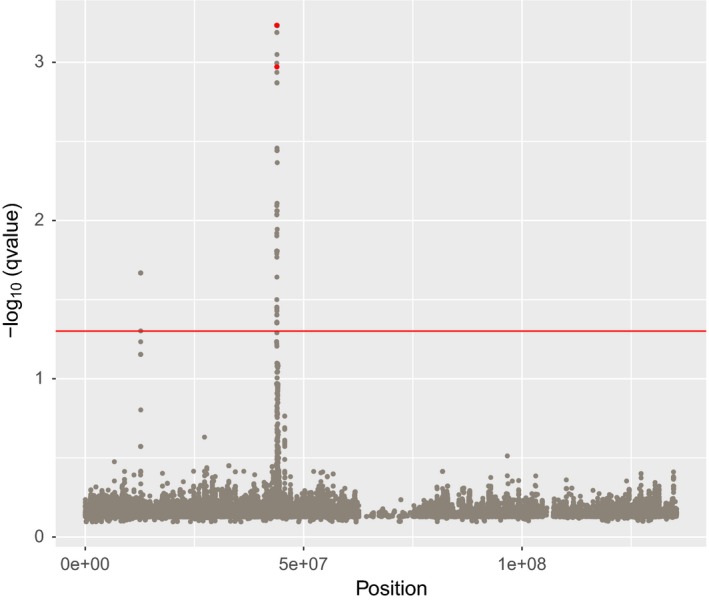
Manhattan plot showing the *q*‐values for each marker (with G‐ or Wald‐Score > 6) on chromosome 23 of Moroccan sheep associated with annual precipitation as calculated in samβada in a univariate mode. Points in red correspond to models involving two nonsynonymous SNPs (ss1208941124 and ss1208941157) in the MC5R gene (ss1208941124 having the lowest q‐value of the two). The red horizontal bar shows a significance threshold of 0.05 [Colour figure can be viewed at wileyonlinelibrary.com]

When investigating samβada's results, a significant peak around position 4.38e7 was observed on chromosome 23 in association with annual precipitation (Figure 2). Within this genomic region, two SNPs (i.e., ss1208941124 at position 23: 43867891 and ss1208941157 at position 23: 43869831) were found to be nonsynonymous for the gene *MC5R* (melanocortin 5 receptor) and in strong LD (*r* ^2^ = 0.97).

**Figure 3:**
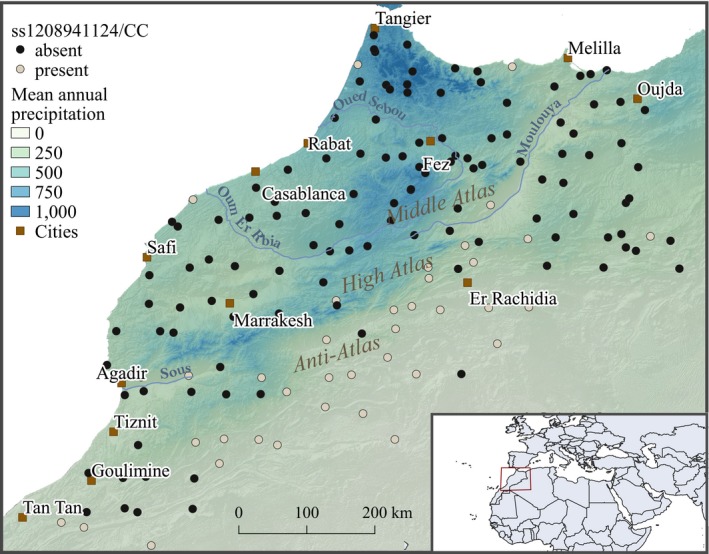
Spatial occurrence of the CC genotype for SNP ss1208941124. In the background, the shaded topography with mean annual precipitation (given in [mm/year]) is displayed [Colour figure can be viewed at wileyonlinelibrary.com]

Given such a high LD, the spatial distribution of these markers is almost identical (except for one individual; data not shown), and only ss1208941124 is illustrated (Figure 3). For this locus, genotype CC is very frequent in the northern part of Morocco, where annual precipitation is on average high (reaching values of 1,000 mm/year), while being almost absent in the south (at the Sahara Desert's gate where precipitation is as low as 50 mm/year).

### 3.3 Lidia cattle in Spain

#### 3.3.1 Population structure

**Figure 4:**
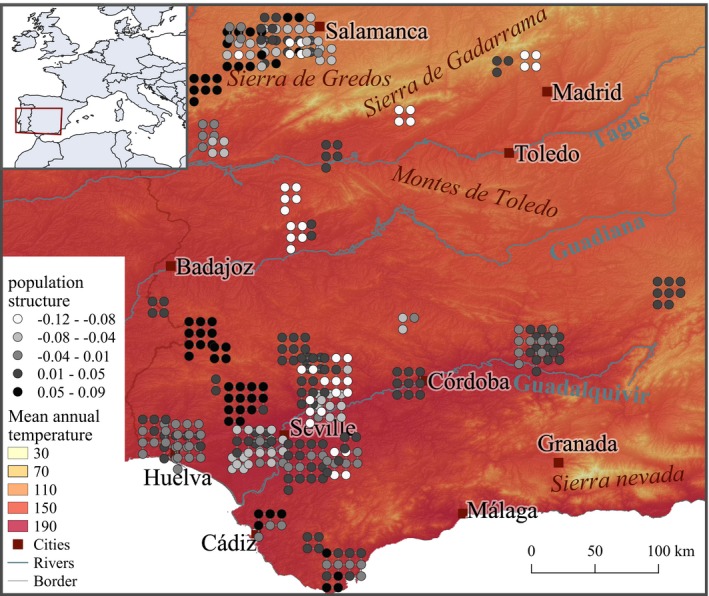
Spatial distribution of the Lidia cattle population structure according to the scores of the first principal component, with a shaded relief and mean annual temperature [°C * 10] as background, as provided in the WorldClim database. Due to overlaps, close points are scattered around the farm [Colour figure can be viewed at wileyonlinelibrary.com]

The variance explained by the first three components of the PCA was 0.049, 0.029 and 0.024, respectively. In this case, the first principal component is likely to represent population structure, given the difference in variance observed between PC 1 and 2, and in accordance with what has been previously observed in between European cattle breeds (e.g., Orozco‐terWengel et al., 2015). Geographically, genetic clusters composed of either single or groups of proximately located farms were identified (e.g., south from Badajoz), although no wider spatial pattern was evident (e.g., north–south gradient) (Figure 4).

#### 3.3.2 Genotype‐environment associations

**Figure 5:**
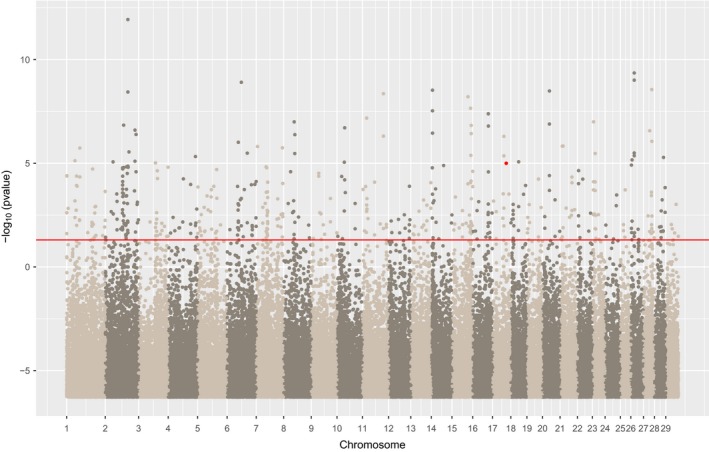
Manhattan plot of the Lidia cattle study, showing the *p*‐values with Bonferroni correction as derived from the samβada models involving mean annual temperature and one population variable. The red point corresponds to SNP ARS‐BFGL‐NGS‐106879, located 30,000 base pairs apart from the HSPB8 gene [Colour figure can be viewed at wileyonlinelibrary.com]

Several narrow peaks were observed in the models involving mean annual temperature (i.e., bio1 bioclim variable) (Figure 5). In particular, the Ensembl query revealed the SNP ARS‐BFGL‐NGS‐106879 (at position 17: 56127482) to be located ~ 30,000 base pairs from the gene *HSPB8* (heat shock protein family B [small] member 8).

**Figure 6:**
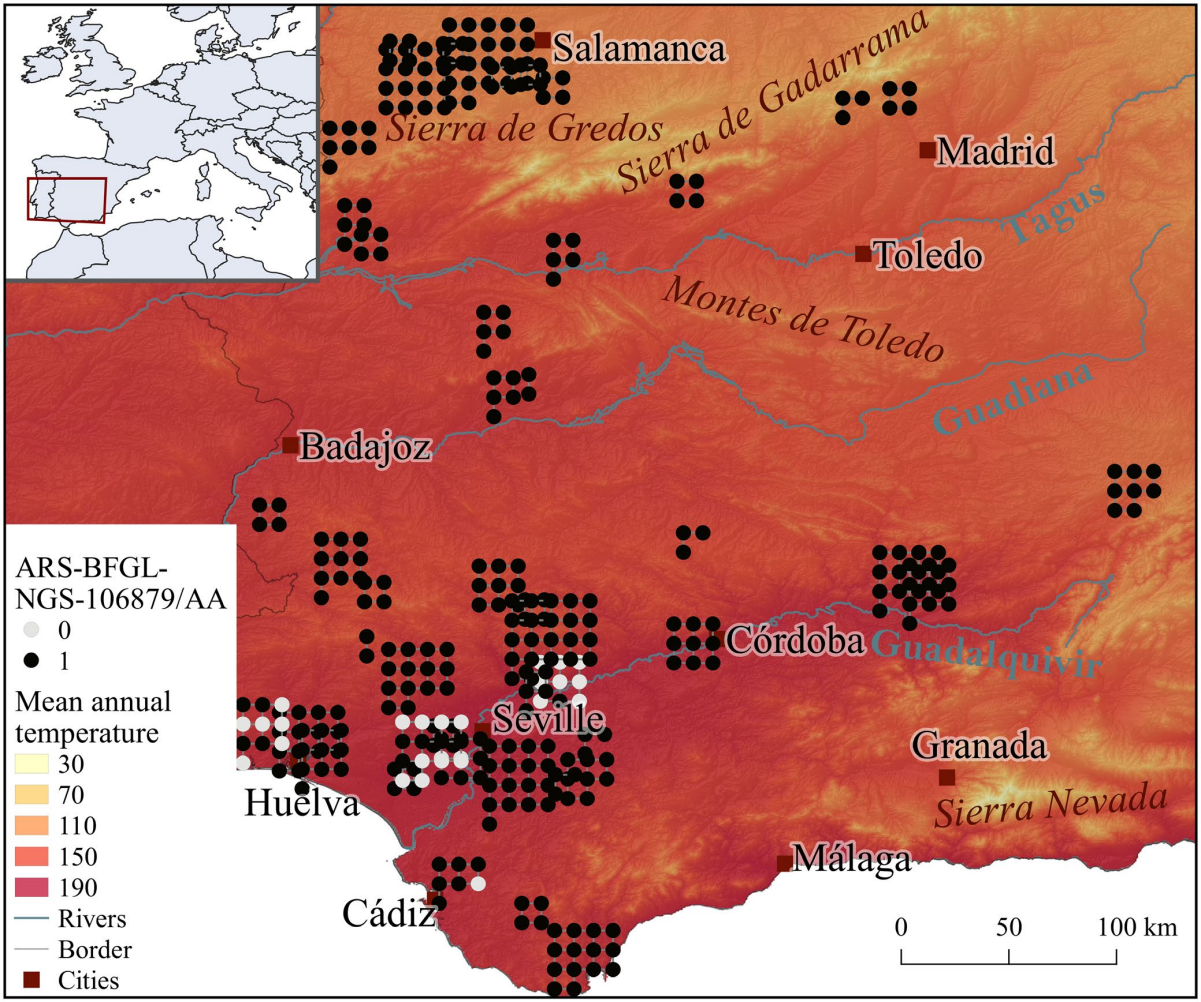
Presence–absence of the AA genotype of SNP ARS‐BFGL‐NGS‐106879 reported with shaded relief and mean annual temperature [°C * 10]) as background. Due to overlaps, close points are scattered around the farm [Colour figure can be viewed at wileyonlinelibrary.com]

Spatial occurrence of genotype AA from ARS‐BFGL‐NGS‐106879 appears to be related to mean annual temperature (Figure 6). More specifically, this genotype is geographically widespread in the study area, except for 23 individuals found in different farms from the Guadalquivir valley, a region with temperature reaching 36°C during the hottest month of the year. Importantly, however, when comparing Figures 4 and 6 it can be seen that the genotype distribution does not match the prevailing population structure; hence, this result is independent of the calculated population structure present within the breed.

## 4 DISCUSSION

### 4.1 Role of the package

We have provided a demonstration of R.SamBada, encompassing the entire pipeline analysis from *pre*‐ to *post hoc* processing, following the classical samβada analysis pathway, but much more efficiently. R.SamBada helps saving user's time for preparing input files thanks to newly built functions, as well as computing time through better integration of population structure and automated split of computations on parallel cores. Additionally, it provides a standardized processing chain, thus facilitating reproducibility.

Moreover, part of the pre‐ and postprocessing chain can possibly be coupled with other software used in landscape genomics and more generally with software designed to detect signature of selection. For example, the postprocessing function *plotResultInteractive* could be used with any type of outputs as long as its structure is similar to the returned value of *prepareOutput* (i.e., columns indicating the position of the SNP as well as the p‐value associated with the corresponding genotype; refer to the package documentation for more detail).

### 4.2 Case studies

#### 4.2.1 Sheep in Morocco

Two of the SNPs on chromosome 23 associated with precipitation (ss1208941124 and ss1208941157) are nonsynonymous variants located within the *MC5R* gene. Although understudied in sheep, this gene has been reported to be linked to a wide range of physiological functions in different mammal species, including regulation of food intake and sebum secretion (Switonski, Mankowska, & Salamon, 2013). Wax secretion is of particular interest with respect to precipitation; indeed, sebaceous secretions in Merino sheep have been found to hinder *Dermatophilus dermatonomous* infection (Roberts, 1963), a skin disease affecting many domestic and wild animal species that can be lethal in extreme cases. In the same breed, Dermatophilosis outbreaks have been found to be linked with exceptionally rainy years (Yeruham, Elad, & Nyska, 1995). Thus, the secretion of wax could play an important role in protecting sheep against rainy weather, consistent with its environmental relationship with annual precipitation here.

#### 4.2.2 Lidia cattle

The SNP ARS‐BFGL‐NGS‐106879 is associated with mean annual temperature and located in the vicinity of the gene *HSPB8*. This gene is thought to code for a chaperone protein, which is upregulated in presence of heat and other environmental stress, and exerts an important cytoprotective role (Verma et al., 2016). In cattle, this gene was found to be associated with heat tolerance in both crossbred and pure *Bos indicus* Sahiwal in India (Sengar et al., 2018; Verma et al., 2016) that can suggest its putative involvement with adaptation to heat tolerance in Lidia cattle as well.

This SNP lies at ~30 Kbp outside the *HSPB8* coding region, either suggesting the SNP to be in LD with some adaptive variant within the gene or to possibly have an important regulatory effect on transcription. However, considering the relatively low average LD between loci at 30Kbp‐distance (computed *r* ^2^ in this region = 0.2), the existence of a significant variant within the gene is unlikely. In contrast, such a distance would suggest more likely this SNP to be involved in regulatory processes; indeed, according to Brodie, Azaria, and Ofran (2016), large insertions/deletions with regulative roles can be found as far as 2Mbp around a gene and associated with nearby SNPs.

### 4.3 Perspectives

R.SamBada represents a step forward in facilitating the chain of processes required to implement a landscape genomics study. However, several further improvements could be implemented in the future. For example, the query based on the Ensembl database requires a reference genome for the species under investigation, which remains relatively uncommon for nonmodel species. It would therefore be very useful to further develop functions performing a BLAST alignment (Johnson et al., 2008) and see if any match can be found with orthologous genes from related species where genomes have been produced.

In addition, functionalities could be augmented to help the user define ad hoc QC thresholds. For instance, a function allowing species‐specific estimation of LD in order to better calibrate the pruning applied before computing the PCA would be useful. Furthermore, R.SamBada currently only implements basic QC of genetic data (MAF, LD, missingness) and does not test for other useful checks (e.g., Identity By Descent – IBD – or Hardy–Weinberg Equilibrium – HWE). However, such controls can easily be performed with dedicated software like plink (C.C. Chang et al., 2015) or vcftools (Danecek et al., 2011) before entering samβada's r‐pipeline. Moreover, samβada is one among several software solutions to detect selection signatures in a spatial context and can be used in combination with other packages like LFMM (Caye et al., 2019), BayEnv (Günther & Coop, 2013) or both (Stucki et al., 2017) in order to compare the results obtained. Further functionalities could be developed to ease the computation and comparison with those methods.

Finally, it is important to keep in mind that landscape genomic approaches such as samβada implement an explanatory analysis which allows rapid identification of candidate genes, but lacks a validation procedure, meaning that derived hypotheses need to be further tested (e.g., through investigation of variant effect on protein tertiary structure and function or through laboratory experiments).

## AUTHOR CONTRIBUTIONS

S.Dur. wrote the major part of the R‐package with the help of O.S., E.V. and S.S. on specific points. In particular, the new functionalities of the C++ code were developed by S.S. N.S. wrote the sections of the manuscript dedicated to the Lidia cattle case study with the help of S.Dun. who was responsible of the Lidia samples availability. K.L. performed most of the analysis related to the Moroccan sheep case study and elaborated part of the related text. S.Dur. wrote the rest of the manuscript with the help of all authors. S.J. conceived and supervised the project. P.O., M.W.B. and S.J. revised the manuscript.

## SOFTWARE AVAILABILITY

R.SamBada package is available in the r CRAN package repository and on GitHub (github.com/SolangeD/R.SamBada).

## Data Availability

The Moroccan sheep data set is available at https://projects.ensembl.org/nextgen/ population MODA. The Lidia cattle data set is accessible from FigShare: https://doi.org/10.6084/m9.figshare.5394895.v4 (only Spanish samples included in the analysis).
