# Supplementary material for: Rapid identification and interpretation of gene–environment associations using the new R.SamBada landscape genomics pipeline
Source: Mol Ecol Resour. 2019 Jun 24;19(5):1355–65. doi: 10.1111/1755-0998.13044 (PMC6790591; doi:10.1111/1755-0998.13044)
Supplement: Supplementary file 1 [file MEN-19-1355-s001.docx]

**Supplemental Information for:**

**Rapid identification and interpretation of gene-environmenta associations using the new R.SamBada landscape genomics pipeline**

Solange Duruz, Natalia Sevane, Oliver Selmoni, Elia Vajana, Kevin Leempoel, Sylvie Stucki, Pablo Orozco-terWengel, Estelle Rochat, Susana Dunner, the NEXTGEN Consortium, the CLIMGEN Consortium, Michael W. Bruford, Stéphane Joost


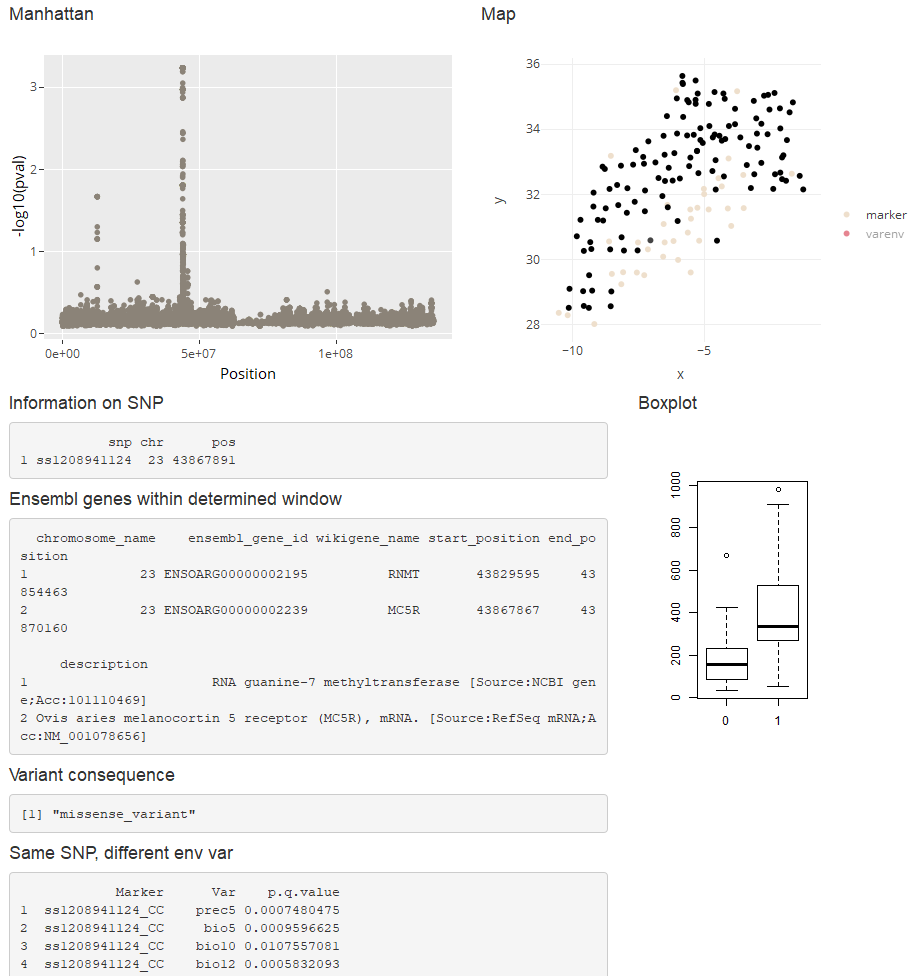


Figure S1: Interactive plot as a result of the function plotResultInteractive. The manhattan plots the q-value of SNPs included in the chromosome 23 of the sheep dataset. In this graph, the genotype ss1208941124_CC was selected, so as to show information on the SNP (position, nearby genes, variant consequence). A map of the genotype (or environmental variable if selected) is available, as well as a boxplot showing the distribution of the environmental variable for the two classes of individuals (absence versus presence of genotype CC). Finally, the window also displays the results of the same SNP included in other models.
